# Febrile illness in high-risk children: a prospective, international observational study

**DOI:** 10.1007/s00431-022-04642-1

**Published:** 2022-10-15

**Authors:** Fabian J. S. van der Velden, Gabriella de Vries, Alexander Martin, Emma Lim, Ulrich von Both, Laura Kolberg, Enitan D. Carrol, Aakash Khanijau, Jethro A. Herberg, Tisham De, Rachel Galassini, Taco W. Kuijpers, Federico Martinón-Torres, Irene Rivero-Calle, Clementien L. Vermont, Nienke N. Hagedoorn, Marko Pokorn, Andrew J. Pollard, Luregn J. Schlapbach, Maria Tsolia, Irini Elefhteriou, Shunmay Yeung, Dace Zavadska, Colin Fink, Marie Voice, Werner Zenz, Benno Kohlmaier, Philipp K. A. Agyeman, Effua Usuf, Fatou Secka, Ronald de Groot, Michael Levin, Michiel van der Flier, Marieke Emonts, PERFORM consortium, Michael Levin, Aubrey Cunnington, Tisham De, Jethro Herberg, Myrsini Kaforou, Victoria Wright, Lucas Baumard, Evangelos Bellos, Giselle D’Souza, Rachel Galassini, Dominic Habgood-Coote, Shea Hamilton, Clive Hoggart, Sara Hourmat, Heather Jackson, Ian Maconochie, Stephanie Menikou, Naomi Lin, Samuel Nichols, Ruud Nijman, Oliver Powell, Ivonne Pena Paz, Priyen Shah, Ching-Fen Shen, Ortensia Vito, Clare Wilson, Amina Abdulla, Ladan Ali, Sarah Darnell, Rikke Jorgensen, Sobia Mustafa, Salina Persand, Molly M. Stevens, Nayoung Kim, Eunjung Kim, Katy Fidler, Julia Dudley, Vivien Richmond, Emma Tavliavini, Ching-Fen Shen, Ching-Chuan Liu, Shih-Min Wang, Federico Martinón-Torres, Antonio Salas, Fernando Álvez González, Cristina Balo Farto, Ruth Barral-Arca, María Barreiro Castro, Xabier Bello, Mirian Ben García, Sandra Carnota, Miriam Cebey-López, María José Curras-Tuala, Carlos Durán Suárez, Luisa García Vicente, Alberto Gómez-Carballa, Jose Gómez Rial, Pilar Leboráns Iglesias, Federico Martinón-Torres, Nazareth Martinón-Torres, José María Martinón Sánchez, Belén Mosquera Pérez, Jacobo Pardo-Seco, Lidia Piñeiro Rodríguez, Sara Pischedda, Sara Rey Vázquez, Irene Rivero Calle, Carmen Rodríguez-Tenreiro, Lorenzo Redondo-Collazo, Miguel Sadiki Ora, Antonio Salas, Sonia Serén Fernández, Cristina Serén Trasorras, Marisol Vilas Iglesias, Dace Zavadska, Anda Balode, Arta Bārzdiņa, Dārta Deksne, Dace Gardovska, Dagne Grāvele, Ilze Grope, Anija Meiere, Ieva Nokalna, Jana Pavāre, Zanda Pučuka, Katrīna Selecka, Aleksandra Rudzāte, Dace Svile, Urzula Nora Urbāne, Effua Usuf, Kalifa Bojang, Syed M. A. Zaman, Fatou Secka, Suzanne Anderson, Anna RocaIsatou Sarr, Momodou Saidykhan, Saffiatou Darboe, Samba Ceesay, Umberto D’alessandro, Henriëtte A. Moll, Clementien L Vermont, Dorine M. Borensztajn, Nienke N. Hagedoorn, Chantal Tan, Joany Zachariasse, W Dik, Philipp KA Agyeman, Christoph Berger, Eric Giannoni, Martin Stocker, Klara M Posfay-Barbe, Ulrich Heininger, Sara Bernhard-Stirnemann, Anita Niederer-Loher, Christian R. Kahlert, Giancarlo Natalucci, Christa Relly, Thomas Riedel, Christoph Aebi, Luregn J Schlapbach, Enitan D Carrol, Elizabeth Cocklin, Rebecca Jennings, Joanne Johnston, Aakash Khanijau, Simon Leigh, Nadia Lewis-Burke, Karen Newall, Sam Romaine, Maria Tsolia, Irini Eleftheriou, Maria Tambouratzi, Antonis Marmarinos, Marietta Xagorari, Kelly Syggelou, Colin Fink, Marie Voice, Leo Calvo-Bado, Werner Zenz, Benno Kohlmaier, Nina A. Schweintzger, Manfred G. Sagmeister, Daniela S. Kohlfürst, Christoph Zurl, Alexander Binder, Susanne Hösele, Manuel Leitner, Lena Pölz, Glorija Rajic, Sebastian Bauchinger, Hinrich Baumgart, Martin Benesch, Astrid Ceolotto, Ernst Eber, Siegfried Gallistl, Gunther Gores, Harald Haidl, Almuthe Hauer, Christa Hude, Markus Keldorfer, Larissa Krenn, Heidemarie Pilch, Andreas Pfleger, Klaus Pfurtscheller, Gudrun Nordberg, Tobias Niedrist, Siegfried Rödl, Andrea Skrabl-Baumgartner, Matthias Sperl, Laura Stampfer, Volker Strenger, Holger Till, Andreas Trobisch, Sabine Löffler, Shunmay Yeung, Juan Emmanuel Dewez, Martin Hibberd, David Bath, Alec Miners, Ruud Nijman, Elizabeth Fitchett, Ronald de Groot, Michiel van der Flier, Marien I. de Jonge, Koen van Aerde, Wynand Alkema, Bryan van den Broek, Jolein Gloerich, Alain J. van Gool, Stefanie Henriet, Martijn Huijnen, Ria Philipsen, Esther Willems, G.P.J.M. Gerrits, M. van Leur, J. Heidema, L. de Haan, C.J. Miedema, C. Neeleman, C.C. Obihara, G.A. Tramper-Stranders, Andrew J. Pollard, Rama Kandasamy, Stéphane Paulus, Michael J. Carter, Daniel O’Connor, Sagida Bibi, Dominic F. Kelly, Meeru Gurung, Stephen Thorson, Imran Ansari, David R. Murdoch, Shrijana Shrestha, Zoe Oliver, Marieke Emonts, Emma Lim, Lucille Valentine, Karen Allen, Kathryn Bell, Adora Chan, Stephen Crulley, Kirsty Devine, Daniel Fabian, Sharon King, Paul McAlinden, Sam McDonald, Anne McDonnell, Ailsa Pickering, Evelyn Thomson, Amanda Wood, Diane Wallia, Phil Woodsford, Frances Baxter, Ashley Bell, Mathew Rhodes, Rachel Agbeko, Christine Mackerness, Bryan Baas, Lieke Kloosterhuis, Wilma Oosthoek, Tasnim Arif, Joshua Bennet, Kalvin Collings, Ilona van der Giessen, Alex Martin, Aqeela Rashid, Emily Rowlands, Gabriella de Vries, Fabian van der Velden, Joshua Soon, Lucille Valentine, Mike Martin, Ravi Mistry, Ulrich von Both, Laura Kolberg, Manuela Zwerenz, Judith Buschbeck, Christoph Bidlingmaier, Vera Binder, Katharina Danhauser, Nikolaus Haas, Matthias Griese, Tobias Feuchtinger, Julia Keil, Matthias Kappler, Eberhard Lurz, Georg Muench, Karl Reiter, Carola Schoen, François Mallet, Karen Brengel-Pesce, Alexandre Pachot, Marine Mommert, Marko Pokorn, Mojca Kolnik, Katarina Vincek, Tina Plankar Srovin, Natalija Bahovec, Petra Prunk, Veronika Osterman, Tanja Avramoska, Taco Kuijpers, Ilse Jongerius, J. M. van den Berg, D. Schonenberg, A. M. Barendregt, D. Pajkrt, M. van der Kuip, A. M. van Furth, Evelien Sprenkeler, Judith Zandstra, G. van Mierlo, J. Geissler

**Affiliations:** 1 Paediatric Immunology, Infectious Diseases & Allergy, Great North Children’s Hospital, Newcastle Upon Tyne Hospitals NHS Foundation Trust, Newcastle upon Tyne, UK; 2 Translational and Clinical Research Institute, Newcastle University, Newcastle upon Tyne, UK; 3 Department of General Paediatrics, Erasmus MC-Sophia Children’s Hospital, Rotterdam, The Netherlands; 4 Population Health Sciences Institute, Newcastle University, Newcastle upon Tyne, UK; 5 Division Paediatric Infectious Diseases, Dr. Von Hauner Children’s Hospital, University Hospital LMU Munich, Munich, Germany; 6 Institute of Infection, Veterinary and Ecological Sciences, University of Liverpool, Liverpool, UK; 7 Alder Hey Children’s NHS Foundation Trust, Liverpool, UK; 8 Section of Paediatric Infectious Disease, Wright-Fleming Institute, Imperial College London, London, UK; 9 Department of Pediatric Immunology, Rheumatology and Infectious Diseases, Amsterdam University Medical Center, Location Academic Medical Center, University of Amsterdam, Amsterdam, The Netherlands; 10 Pediatrics Department, Translational Pediatrics and Infectious Diseases, Hospital Clínico Universitario de Santiago, Santiago de Compostela, Spain; 11 University Children’s Hospital, University Medical Centre Ljubljana, Ljubljana, Slovenia; 12 Oxford Vaccine Group, Department of Paediatrics, University of Oxford, Oxford, UK; 13 Neonatal and Pediatric Intensive Care Unit, Children’s Research Center, University Children’s Hospital Zürich, University of Zürich, Zurich, Switzerland; 14 2nd Department of Pediatrics, National and Kapodistrian University of Athens, Children’s Hospital ‘P, and A. Kyriakou’, Athens, Greece; 15 Clinical Research Department, Faculty of Infectious and Tropical Disease, London School of Hygiene and Tropical Medicine, London, UK; 16 Department of Pediatrics, Rīgas Stradina Universitāte, Children’s Clinical University Hospital, Riga, Latvia; 17 Micropathology Ltd, University of Warwick, Warwick, UK; 18 Department of Pediatrics and Adolescent Medicine, Division of General Pediatrics, Medical University of Graz, Graz, Austria; 19 Pediatric Infectious Diseases and Immunology, Amalia Children’s Hospital, Radboud University Medical Center, Nijmegen, The Netherlands; 20 NIHR Newcastle Biomedical Research Centre, Newcastle Upon Tyne Hospitals NHS Trust and Newcastle University, Newcastle upon Tyne, UK; 21 Department of Pediatrics, Division of Pediatric Infectious Diseases & Immunology, Erasmus MC-Sophia Children’s Hospital, Rotterdam, The Netherlands; 22 Pediatric Infectious Diseases and Immunology, Wilhelmina Children’s Hospital University Medical Center Utrecht, Utrecht, The Netherlands; 23 Department of Pediatrics, Inselspital, Bern University Hospital, University of Bern, Bern, Switzerland; 24 Medical Research Council Unit, Serrekunda, The Gambia; 25 Grupo de Genetica, Vacunas, Infecciones y Pediatria, Instituto de Investigacion Sanitaria de Santiago, Universidad de Santiago, Santiago de Compostela, Spain; 26 Consorcio Centro de Investigacion Biomedicaen Red de Enfermedades Respiratorias (CIBERES), Madrid, Spain

**Keywords:** Immunocompromised, Paediatric, Fever, Infection, Antibiotics

## Abstract

To assess and describe the aetiology and management of febrile illness in children with primary or acquired immunodeficiency at high risk of serious bacterial infection, as seen in emergency departments in tertiary hospitals. Prospective data on demographics, presenting features, investigations, microbiology, management, and outcome of patients within the ‘Biomarker Validation in HR patients’ database in PERFORM, were analysed. Immunocompromised children (< 18 years old) presented to fifteen European hospitals in nine countries, and one Gambian hospital, with fever or suspected infection and clinical indication for blood investigations. Febrile episodes were assigned clinical phenotypes using the validated PERFORM algorithm. Logistic regression was used to assess the effect size of predictive features of proven/presumed bacterial or viral infection. A total of 599 episodes in 482 children were analysed. Seventy-eight episodes (13.0%) were definite bacterial, 67 episodes probable bacterial (11.2%), and 29 bacterial syndrome (4.8%). Fifty-five were definite viral (9.2%), 49 probable viral (8.2%), and 23 viral syndrome (3.8%). One hundred ninety were unknown bacterial or viral infections (31.7%), and 108 had inflammatory or other non-infectious causes of fever (18.1%). Predictive features of proven/presumed bacterial infection were ill appearance (OR 3.1 (95% CI 2.1–4.6)) and HIV (OR 10.4 (95% CI 2.0–54.4)). Ill appearance reduced the odds of having a proven/presumed viral infection (OR 0.5 (95% CI 0.3–0.9)). A total of 82.1% had new empirical antibiotics started on admission (*N* = 492); 94.3% proven/presumed bacterial (*N* = 164), 66.1% proven/presumed viral (*N* = 84), and 93.2% unknown bacterial or viral infections (*N* = 177). Mortality was 1.9% (*N* = 11) and 87.1% made full recovery (*N* = 522).

*   Conclusion*: The aetiology of febrile illness in immunocompromised children is diverse. In one-third of cases, no cause for the fever will be identified. Justification for standard intravenous antibiotic treatment for every febrile immunocompromised child is debatable, yet effective. Better clinical decision-making tools and new biomarkers are needed for this population.

**Table:** 

**What is Known:** *• Immunosuppressed children are at high risk for morbidity and mortality of serious bacterial and viral infection, but often present with fever as only clinical symptom.* *• Current diagnostic measures in this group are not specific to rule out bacterial infection, and positivity rates of microbiological cultures are low.*	
**What is New:** *• Febrile illness and infectious complications remain a significant cause of mortality and morbidity in HR children, yet management is effective.* *• The aetiology of febrile illness in immunocompromised children is diverse, and development of pathways for early discharge or cessation of intravenous antibiotics is debatable, and requires better clinical decision-making tools and biomarkers.*

**Supplementary Information:**

The online version contains supplementary material available at 10.1007/s00431-022-04642-1.

## Introduction

Complex comorbidities render a growing number of children who attend the emergency department (ED) at increased risk of infection [1]. This includes children with primary (PID) or acquired immunodeficiencies, but also those who are dependent on total parenteral nutrition (TPN) with central venous lines. They are at high risk (HR) for serious bacterial infection (SBI) and life-threatening infectious complications [1, 2]. Some of these children are neutropenic. SBI during febrile neutropenia (FN) is a medical emergency associated with significant morbidity and mortality if left untreated [3, 4]. One-third of neutropenic episodes in paediatric patients on cancer treatment or during haematopoietic stem cell transplant (HSCT) are associated with fever [5].

Differentiating viral, bacterial, and inflammatory illnesses on admission is challenging in HR patients: clinical syndromes are often non-specific, and at least 36–48 h is needed to culture microorganisms [4, 6]. Because the risk of having SBI is significant [4, 7], immunocompromised patients with febrile illness are virtually always admitted for intravenous antibiotic treatment, awaiting microbiological results, yet only around 20% will have a microbiologically documented infection [3, 8–10]. 41.3–62.3% will have no cause identified [11–14].

This approach has led to a significant reduction in mortality and morbidity [15], but consequently antibiotic overuse, increased risk of antimicrobial resistance, and prolonged hospitalisation. Fever accounts for 60.2% of emergency department (ED) attendance in paediatric cancer [16] and is a significant burden for caregivers [17] and healthcare systems. Commonly used biomarkers such as C-reactive protein (CRP) and procalcitonin aid the diagnostic process, but are often not sensitive enough to rule out bacterial infection [18, 19]. It is suspected that a large proportion of HR fever has a viral aetiology, is drug-induced, or is caused by an underlying disease [20–25].

We describe the aetiology and management of fever in immunocompromised children, and assess the risk of bacterial and viral infections in a mixture of immunocompromised patients as seen by paediatricians in tertiary healthcare centre EDs, where they present primarily with fever.

## Material and methods

This prospective, international, multicentre, observational study assessed children recruited to the ‘Biomarker Validation in HR patients’ cohort within Personalised Risk assessment in Febrile illness to Optimise Real-life Management across the European Union (PERFORM) between 2 June 2016 and 31 December 2019.

### Participants

Children, < 18 years of age, immunocompromised due to primary or secondary immunodeficiency, were eligible upon presentation to ED, ward, or intensive care unit (PICU) admission from their usual place of residence for community-acquired infections, or from wards or PICU for suspected hospital-acquired infections with (history of) fever (< 72 h prior to admission, *T* ≥ 38.0 °C) or suspected infection, and clinical indication for blood investigations as per treating clinician’s decision. Participants could have multiple episodes, with a 2-week minimum between the end and start of consecutive episodes. They were recruited between 2 June 2016 and 31 December 2019.

Participants were recruited from sixteen tertiary centres in ten countries: four each from the United Kingdom (UK) and the Netherlands, and one each from Austria, the Gambia, Germany, Greece, Latvia, Slovenia, Spain, and Switzerland.

### Data collection

We collected in-depth clinical data on standardised forms, including clinical symptoms, laboratory results, management, clinical syndromes, 28-day follow-up by phone call or in outpatient clinic, severity, and mortality. Regular data quality control was performed on the data in the online database. Missing values, outliers, abnormal laboratory results, and dates were double checked to ensure they were correct for the respective episode.

### Study outcomes

All episodes were assigned final phenotypes using the validated algorithm in the PERFORM protocol [26] (Supplementary Information Fig. S1), previously described by Nijman et al. [27], and assigned one of eleven phenotypes: definite bacterial, probable bacterial, bacterial syndrome, unknown bacterial/viral, viral syndrome, probable viral, definite viral, trivial, other infection, uncertain infection/inflammation, or inflammatory syndrome. Episodes assigned definite bacterial, probable bacterial, or unknown bacterial/viral phenotypes could also have viral or fungal co-infection identified. Phenotypes for all episodes were reviewed by two experienced paediatricians before definite assignment. In case of disagreement or uncertainty by the assigning paediatricians, episodes were anonymously discussed with paediatricians from the other sites within the consortium before assigning the final phenotype.

To evaluate determinants of bacterial and viral infection, we combined definite bacterial, probable bacterial, and bacterial syndromes to a proven/presumed bacterial infection group, and definite viral, probable viral, and viral syndromes to a proven/presumed viral infection group. The grouping was performed as these children would receive the same initial treatment and management, and it would allow us to assess the wider spectrum of bacterial and viral diseases. The definite bacterial phenotype could only be assigned if the bacterium was isolated from a sterile site.

First, we described our cohort and compared clinical features of proven/presumed bacterial, and proven/presumed viral groups versus the other phenotypes. Neutropenia was defined as absolute neutrophil count (ANC) < 0.5 × 10^9^/L or < 1.0 × 10^9^/L but expected < 0.5 × 10^9^/L within 48 h, or, if no ANC available, white cell count < 1.0 × 10^9^/L [28] and lymphopenia defined as lymphocyte count < 1.0 × 10^9^/L. Second, we described microbiology results and empirical antimicrobial management, utilising the AWaRe classification [29], categorising antibiotics in ‘access’, ‘watch’, and ‘reserve’ groups. Last, we described clinical syndromes, severity, and outcome.

### Statistical analysis

Data was analysed using IBM SPSS Statistics, version 27 (Armonk, USA 2020). For descriptive data, absolute frequencies and percentages were used. Data was not normally distributed; non-parametric tests, medians, and interquartile ranges (IQR) were used. The Mann–Whitney *U* tests were used for continuous variables and Pearson’s *χ*^2^ or Fisher’s exact tests for categorical data. To assess the size effect of significantly associated clinical features for proven/presumed bacterial or viral infections, odds ratios (ORs) and 95% confidence intervals (CIs) were calculated using univariate binary logistic regression. Subsequently, multivariate binary logistic regression was performed on variables with significant ORs after univariate binary logistic regression. *P*-values < 0.05 were considered significant.

## Results

A total of 599 episodes in 482 children were analysed. Eighty-four children had multiple febrile episodes, with a maximum of six episodes in one child. A total of 343 episodes were in males (57.3%), and median age at admission was 7.7 years (IQR 4.1–12.8 years). Eight patients were from the Gambia (1.3%).

### Final phenotype diagnoses

**Fig. 1:**
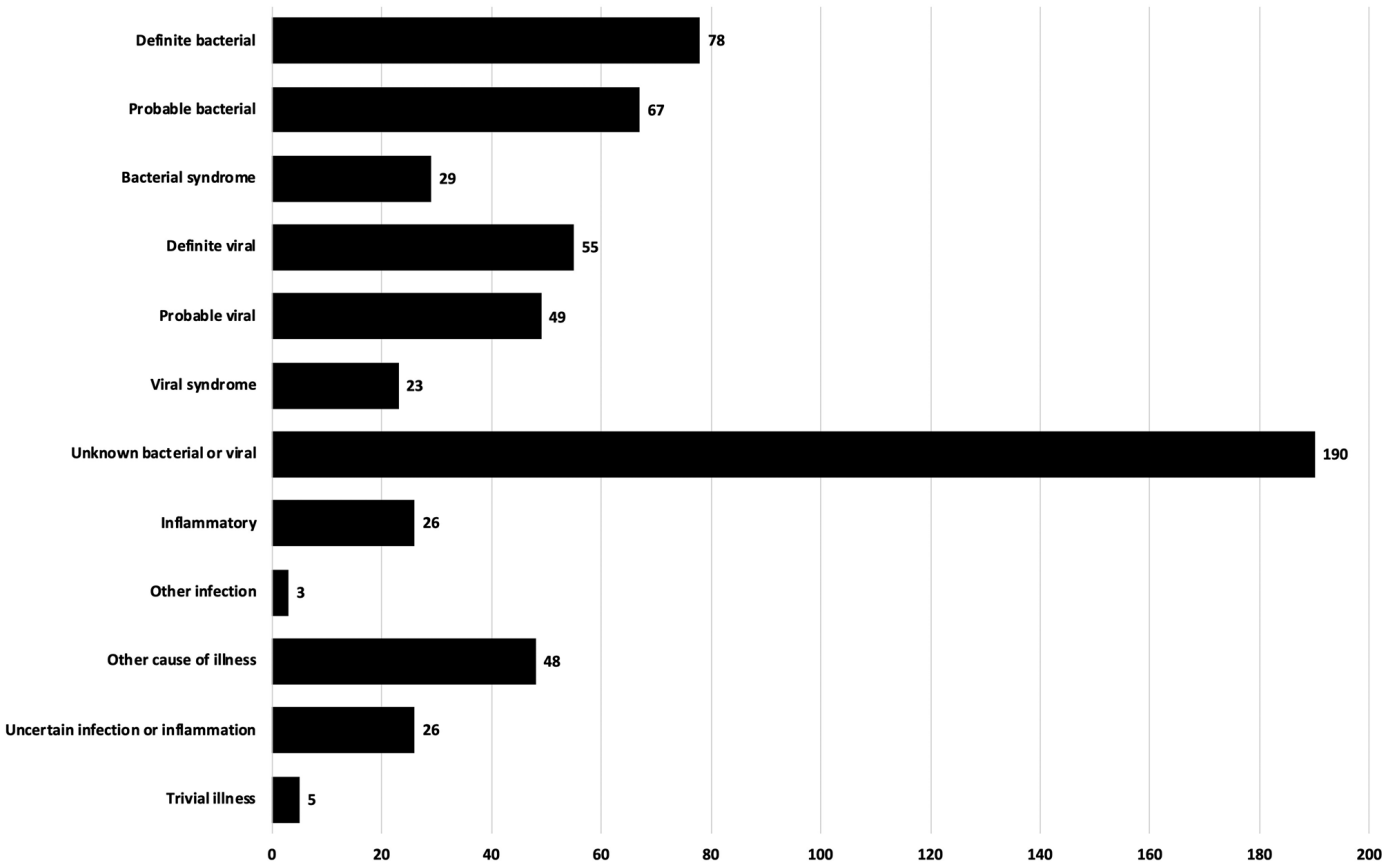
Final phenotypes assigned by episode as per PERFORM protocol (*N* = 599 episodes)

A total of 174 episodes (29.0%) were proven/presumed bacterial, of which 78 were definite bacterial (13.0%). A total of 127 episodes were proven/presumed viral (21.2%), of which 55 were definite viral (9.2%). A total of 190 episodes (31.7%) were unknown bacterial or viral infections (Fig. 1).

### Demographics

Table 1 gives a demographic overview, with detailed data on demographics per phenotype in Supplementary Information Table S1 and underlying conditions in Supplementary Information Table S2. Most common underlying conditions were malignancies in 354 episodes (59.2%), followed by non-malignant haematological disease in 79 (13.2%), and inflammatory disease and PID with 47 episodes (7.8%) each. Of the Gambian patients, 7 had sickle cell disease, and 1 HIV, as an underlying condition.

**Table 1:** Cohort demographics at admission

	**All (*N* = 599)**	**Proven/presumed bacterial (*N* = 174)**	**Proven/presumed viral** **(*N* = 127)**	**Unknown bacterial or viral infection (*N* = 190)**	**Other phenoypes (*N* = 108)**	**Proven/presumed bacterial vs all other phenotypes** **(*p*-value)**	**Proven/presumed viral vs all other phenotypes (*p*-value)**	**Missing values (*N* = 599)**
Male	343 (57.3%)	101 (58.0%)	70 (55.1%)	107 (56.3%)	65 (60.2%)	0.80	0.58	-
Age (years)	7.7 (4.1–12.8)	7.9 (3.5–12.9)	7.2 (4.4–12.2)	6.5 (4.1–11.7)	10.0 (4.6–14.9)	0.91	0.61	-
HSCT patient	69 (11.5%)	15 (8.6%)	19 (15.0%)	15 (7.9%)	20 (18.5%)	0.16	0.17	-
**Underlying condition**								
Malignancy	354 (59.2%)	98 (56.4%)	69 (54.3%)	150 (78.9%)	37 (34.2%)	0.38	0.22	-
Haematological disease	79 (13.2%)	21 (12.1%)	19 (15.0%)	19 (10.0%)	20 (18.5%)	0.60	0.51	-
Inflammatory syndromes	47 (7.8%)	7 (4.0%)	9 (7.1%)	5 (2.6%)	26 (24.1%)	0.03	0.72	-
Primary immunodeficiency	47 (7.8%)	10 (5.7%)	14 (11.0%)	8 (4.2%)	15 (13.9%)	0.22	0.13	-
Solid organ transplant	30 (5.0%)	19 (10.9%)	4 (3.1%)	3 (1.6%)	4 (3.7%)	< 0.001	0.28	-
HIV	8 (1.3%)	6 (3.4%)	0 (0.0%)	0 (0.0%)	2 (1.9%)	0.004	0.21	-
Nephrotic syndrome	6 (1.0%)	3 (1.7%)	1 (0.8%)	1 (0.5%)	1 (0.9%)	0.36	1.00	-
Cystic fibrosis	5 (0.8%)	2 (1.2%)	1 (0.8%)	2 (1.1%)	0 (0.0%)	0.63	1.00	-
Short bowel syndrome	4 (0.7%)	2 (1.2%)	1 (0.8%)	1 (0.5%)	0 (0.0%)	0.58	1.00	-
Other conditions	19 (3.2%)	6 (3.4%)	9 (7.1%)	1 (0.5%)	3 (2.8%)	0.81	0.01	-
**Clinical features**								
Ill appearance	176 (29.4%)	83 (47.7%)	26 (20.5%)	39 (20.5%)	28 (25.9%)	< 0.001	0.01	-
Lifesaving intervention required	54 (9.0%)	26 (14.9%)	8 (6.3%)	9 (4.7%)	11 (10.2%)	0.001	0.23	-
Diarrhoea	45 (7.5%)	14 (8.0%)	10 (7.9%)	9 (4.7%)	12 (11.1%)	0.75	0.86	-
Increased work of breathing	36 (6.0%)	11 (6.3%)	10 (7.9%)	6 (3.2%)	9 (8.3%)	0.84	0.32	-
Vomiting	28 (4.7%)	12 (6.9%)	6 (4.7%)	5 (2.6%)	5 (4.6%)	0.10	0.98	-
Non-blanching rash	15 (2.5%)	6 (3.4%)	2 (1.6%)	2 (1.1%)	5 (4.6%)	0.39	0.75	-
Clinical dehydration	15 (2.5%)	6 (3.4%)	4 (3.1%)	3 (1.6%)	2 (1.9%)	0.39	0.54	-
Seizures	8 (1.3%)	4 (2.3%)	0 (0.0%)	2 (1.1%)	2 (1.9%)	0.24	0.21	-
Meningism	3 (0.5%)	1 (0.6%)	1 (0.8%)	0 (0.0%)	1 (0.9%)	0.87	0.51	-
Vital parameters, age adjusted*								
Tachypnoea	79 (13.2%)	32 (18.4%)	15 (11.8%)	16 (8.4%)	16 (14.8%)	0.02	0.61	151
Bradypnoea	9 (1.5%)	5 (2.9%)	1 (0.8%)	2 (1.1%)	1 (0.9%)	0.13	0.69	151
Low saturation (< 94% in air)	39 (6.5%)	11 (6.3%)	8 (6.3%)	9 (4.7%)	11 (10.2%)	0.91	0.91	123
Tachycardia	189 (31.6%)	69 (39.7%)	35 (37.6%)	63 (33.2%)	22 (20.4%)	0.01	0.28	63
Bradycardia	3 (0.5%)	1 (0.6%)	0 (0.0%)	1 (0.5%)	1 (0.9%)	1.00	1.00	63
Hypotension	48 (8.0%)	14 (8.0%)	11 (8.7%)	15 (7.9%)	8 (7.4%)	0.99	0.76	190
Hypertension	179 (29.9%)	56 (32.2%)	36 (28.3%)	43 (22.6%)	44 (40.7%)	0.43	0.67	190
Prolonged capillary refill time (> 2 s)	16 (2.7%)	8 (5.5%)	2 (1.6%)	5 (2.6%)	1 (0.9%)	0.11	0.54	142
Decreased consciousness (AVPU < A, GCS < = 13)	5 (0.8%)	2 (1.1%)	0 (0.0%)	2 (1.1%)	1 (0.9%)	0.63	0.59	-
Fever (documented/history = > 38.0 °C)	528 (88.1%)	155 (89.1%)	115 (90.6%)	178 (93.7%)	80 (74.1%)	0.65	0.35	-
**Blood investigations**								
Neutropenia	212 (35.4%)	61 (35.1%)	33 (26.0%)	101 (53.2%)	17 (15.7%)	0.87	0.01	3
Lymphopenia	265 (44.2%)	75 (51.7%)	63 (49.6%)	89 (46.8%)	38 (35.2%)	0.63	0.65	103
**Immunomodulating drug use**								
Biologicals	34 (5.7%)	6 (3.4%)	5 (3.9%)	6 (3.2%)	17 (15.7%)	0.13	0.34	-
Ciclosporin	35 (5.8%)	10 (5.7%)	9 (7.1%)	5 (2.6%)	11 (10.2%)	0.95	0.50	-
Colchicine	1 (0.2%)	0 (0.0%)	1 (0.8%)	0 (0.0%)	0 (0.0%)	1.00	0.21	-
Immunoglobulin	39 (6.5%)	9 (5.2%)	10 (7.9%)	8 (4.2%)	12 (11.1%)	0.4	0.48	-
Methotrexate	118 (19.7%)	31 (17.8%)	32 (25.2%)	45 (23.7%)	10 (9.3%)	0.46	0.08	-
Steroids	112 (20.4%)	41 (23.6%)	21 (16.5%)	35 (18.4%)	25 (23.1%)	0.21	0.23	-
Tacrolimus	32 (5.3%)	18 (10.3%)	5 (3.9%)	3 (1.6%)	6 (5.6%)	< 0.001	0.43	-
Other immunomodulating drug	262 (43.7%)	78 (44.8%)	65 (51.2%)	76 (40.0%)	43 (39.8%)	0.73	0.06	-

*GCS*, Glasgow Coma Scale, *HIV* human Immunodeficiency Virus, *HSCT* Haematopoietic Stem Cell Transplant

^*^Age-adjusted vital parameters as per APLS 2017 (> 95th centile or < 5th centile). Data is presented as *N* = episodes (%) or median (IQR). *p*-values were calculated using *χ*^2^, Fisher’s exact, or Mann–Whitney *U* tests as appropriate

In univariate binary logistic regression, the following clinical features at admission were associated with proven/presumed bacterial infections versus all other phenotypes: ill appearance (OR 3.3 (95% CI 2.2–4.7)), tachypnoea (OR 1.8 (95% CI 1.1–2.9)), tachycardia (OR 1.6 (95% CI 1.1–2.4)), requiring a lifesaving intervention (OR 2.5 (95% CI 1.4–4.4)), solid organ transplant recipients (OR 4.7 (95% CI 2.1–9.9)), HIV (OR 7.6 (95% CI 1.5–37.8)), inflammatory disease (OR 0.4 (95% CI 0.2–0.9)), and tacrolimus use (OR 3.4 (95% CI 1.6–6.9)) (Table 2). After the multivariate binary logistic regression, HIV (OR 10.4 (95% CI 2.0–54.4)) and ill appearance (OR 3.1 (95% CI 2.1–4.6)) were the two covariates remaining significant. The model achieved an area under ROC of 0.69 (95% CI 0.65–0.74).

**Table 2:** Univariate and multivariate regression for clinically associated features and proven/presumed bacterial infection and proven/presumed viral infection. Data reported as odds ratio (95% confidence interval)

Clinical features	Univariate logistic regression	Multivariate logistic regression
**Proven/presumed bacterial infection vs other phenotypes**		
Underlying inflammatory condition	0.4 (0.2–0.9)	0.5 (0.2–1.2)
Solid organ transplant	4.7 (2.1–9.9)	3.0 (0.9–10.7)
HIV	7.6 (1.5–37.8)	10.4 (2.0–54.4)
Ill appearance	3.3 (2.2–4.7)	3.1 (2.1–4.6)
Lifesaving intervention required	2.5 (1.4–4.4)	1.4 (0.7–2.6)
Tachypnoea	1.8 (1.1–2.9)	1.3 (0.7–2.2)
Tachycardia	1.6 (1.1–2.4)	1.4 (1.0–2.2)
Tacrolimus use	3.4 (1.6–6.9)	1.5 (0.4–5.1)
**Proven/presumed viral infection vs other phenotypes**		
Other underlying conditions	3.5 (1.4–8.9)	3.9 (1.5–10.0)
Ill appearance	0.6 (0.3–0.9)	0.5 (0.3–0.9)
Neutropenia at admission	1.0 (0.7–1.4)	-

Ill appearance reduced the odds of having a proven/presumed viral infection (OR 0.6 (95% CI 0.3–0.9)), and other underlying conditions increased the odds (OR 3.5 (95% CI 1.4–8.9)) in univariate binary logistic regression (Table 2). Both covariates remained significant after multivariate binary logistic regression, with an achieved area under ROC of 0.58 (95% CI 0.52–0.63).

### Microbiology

Blood cultures were obtained in 563 episodes (94.0%), with a positive yield of 15.1% (*N* = 85). Urine cultures were performed in 165 episodes (27.5%), with a yield of 13.9% (*N* = 23). Polymerase chain reactions, primarily utilised for the detection of viral pathogens, were performed in 258 episodes (43.1%), with a yield of 46.9% (*N* = 121) for any tested pathogen. Rapid antigen testing (*N* = 86, 14.4%), serology (*N* = 61, 10.2%), and tuberculosis testing (*N* = 14, 2.3%) were less frequently performed, with yields of 15.1%, 39.3%, and 7.1%, respectively.

**Fig. 2:**
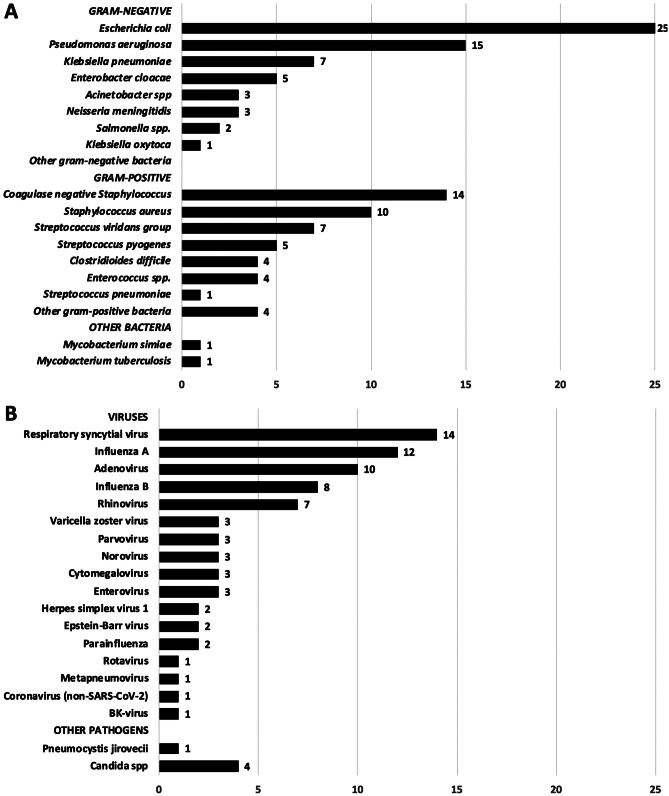
Causative pathogens isolated or detected by episode. In 5 episodes, ≥ 1 causative bacteria were isolated, and in 10 episodes, ≥ 1 virus was detected. **A** Bacteria from blood or other sterile site cultures: other gram-negative: *Burkholderia cepacia* complex, *Citrobacter freundii*, *Delftia acidovorans*, *Fusobacterium nucleatum*, *Haemophilus influenzae* (unspecified), *Serratia marcescens*, all once isolated*.* Other gram-positive: *Corynebacterium* spp., *Kytococcusschroeteri*, *Lactobacillus rhamnosus*

An overview of identified causative pathogens from blood and sterile site cultures is given in Fig. 2. In the definite bacterial group, with positive cultures from sterile sites, 118 bacterial isolates were cultured, of which 67 were gram-negative (56.7%) and 49 were gram-positive (41.5%). Two patients (1.8%) had mycobacterial pathogens identified. Common pathogens in our cohort were *Escherichia coli* (*N* = 25), *Pseudomonas aeruginosa* (*N* = 15), and *Staphylococcus aureus* (*N* = 10). Coagulase-negative staphylococci were the most common gram-positive pathogen, in 14 episodes (Fig. 2A).

For viral pathogens, respiratory syncytial virus (*N* = 14), influenza A (*N* = 12), and adenovirus (*N* = 10) were detected most frequently. In 5 patients, a fungal pathogen was deemed causative (Fig. 2B). Co-infection was documented in 31 episodes, of which 29 were bacterial-viral and 2 bacterial-fungal.

### Empirical antimicrobial treatment

In 492 episodes (82.1%), new empirical antibiotics were started on admission (group by antibiotic class in Table 3, more detailed in Supplementary Information Table S3). A total of 164 proven/presumed bacterial, 84 proven/presumed viral, and 177 unknown bacterial or viral episodes had new antibiotics started on admission. A total of 270 episodes had been treated with non-prophylactic antibiotics within 7 days prior to admission (45.1%). Most given empirical antibiotics were piperacillin-tazobactam (*N* = 197, 40.0%) and teicoplanin (*N* = 115, 23.4%). A total of 440 episodes were started on ‘watch’ antibiotics (73.5%) empirically, and one was started on linezolid, a ‘reserve’ antibiotic, to use only as a last resort drug, according to the World Health Organization AWaRe classification [29].

**Table 3:** Empirical antimicrobials started on admission by episodes (*N* = 599), antibiotics are grouped by class. The total number of antimicrobials exceeds the number of episodes as ≥ 1 antimicrobial could be started for a single episode

Antimicrobial group	*N* = 599	%
Penicillins	**257**	42.9
Glycopeptides	**138**	23.0
Aminoglycosides	**109**	18.2
3rd-generation cephalosporins	**106**	17.7
4th-generation cephalosporins	**71**	11.9
Carbapenems	**41**	6.8
Macrolides	**29**	4.8
Imidazoles	**19**	3.2
2nd-generation cephalosporins	**15**	2.5
Fluroquinolones	**15**	2.5
Other antibiotics	**12**	2.0
Lincosamides	**10**	1.7
DHFR inhibitors	**9**	1.5
1st-generation cephalosporins	**3**	0.5
Amphenicols	**2**	0.3
Oxazolidinones	**1**	0.2
Antivirals	**37**	6.2
Antifungals	**23**	3.8
No antimicrobials	**77**	12.9

Median duration of antibiotic treatment was 7 days (IQR 4–10 days). The proven/presumed bacterial group was treated significantly longer (median 10 days (IQR 7–14 days), *p* < 0.001) and the proven/presumed viral group significantly shorter (median 5 days (IQR 3–8 days), *p* = 0.001). The unknown bacterial or viral group was treated for a median of 5 days (IQR 3–8 days).

### Clinical syndromes

**Fig. 3:**
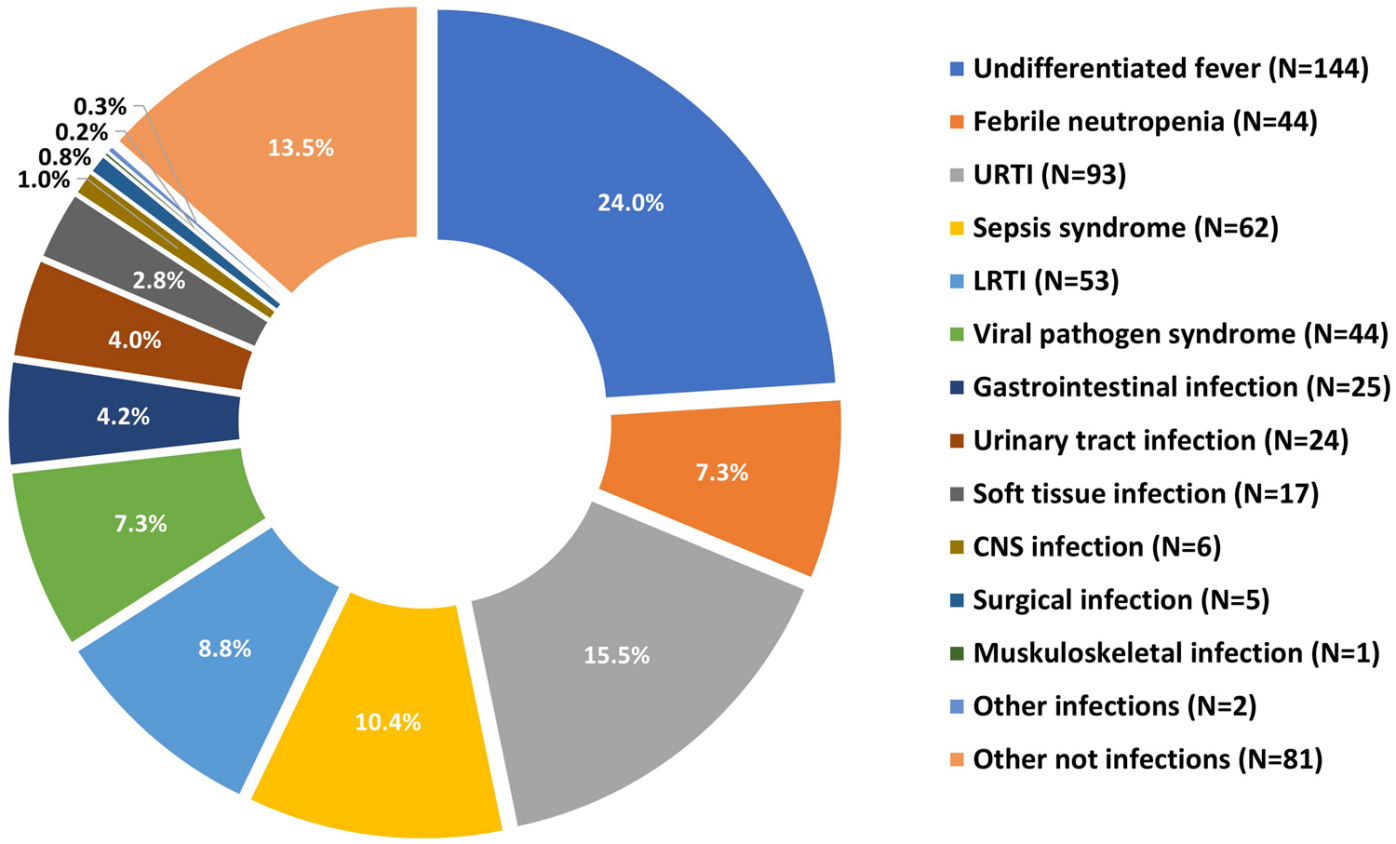
Clinical syndromes by group and by episodes (*N* = 599)

Common foci for febrile illness were upper respiratory tract infections (*N* = 93, 15.5%), and sepsis syndromes (10.4%), who had no specific localised focus for fever, but did have a positive blood culture. A total of 144 episodes were classed as undifferentiated fever, and 42 episodes had febrile neutropenia only, meaning 31.1% of febrile episodes in children at high risk for SBI had no source for the fever identified (Fig. 3, detailed in Supplementary Information Table S4). Eighty-one episodes (13.5%) had non-infectious causes of fever.

### Severity and outcome

Mortality within the febrile illness episode was 1.9% (11 children). Four had a malignancy, three PID, two solid organ transplant, and one sickle cell disease, and one was on prolonged steroids following ischaemic brain injury. Three children died due to viral infection: one had disseminated adenoviraemia, one congenital cytomegalovirus reactivation, and one influenza A whilst developing multi-organ failure due to chemotoxicity from HSCT medication. Two died of sepsis: one *Streptococcus pneumoniae*, one *Candida albicans.* Two children had clinical lower respiratory tract infections but no pathogen isolated. One child died of drug reaction with eosinophilia and systemic symptom (DRESS) syndrome and one of gastrointestinal infection already in palliative care. Two children died of non-infectious cancer-related complications.

In 522 episodes (87.1%), patients made full recovery on the 28-day follow-up, with no significant difference between the proven/presumed bacterial and proven/presumed viral groups. Median length of in-patient stay (LOS) was 5 days (IQR 2–13 days), with longer admissions in the proven/presumed bacterial group (median 7 days (IQR 4–25 days, *p* < 0.001)), and shorter admissions in the proven/presumed viral group (median 2 days (IQR 1–6 days), *p* < 0.001) compared to other phenotypes. PICU admissions were required for 54 episodes (9.0%), which was associated with a proven/presumed bacterial infection (*p* = 0.005). Median admission duration to PICU was 6 days (IQR 2–15). A proven/presumed viral infection was associated with a shorter PICU admission duration (*p* = 0.007) versus other phenotypes. In 17 episodes (2.8%), inotropic support was required, of which 12 had proven/presumed bacterial infections. Sixty-nine episodes required supplemental oxygen (11.5%), 24 (4.0%) non-invasive ventilation, and 27 (4.5%) invasive ventilation. Inotropic support, non-invasive ventilation, and invasive ventilation were associated with proven/presumed bacterial infection when compared to other phenotypes (*p* < 0.001, *p* = 0.001, and *p* = 0.008, respectively).

## Discussion

Our study provides insights into current aetiology and management of febrile illness in immunocompromised children at HR for SBI. In one-third of febrile episodes, no focus for the fever was identified, regardless of advances in laboratory and microbiological investigations. This is lower than previously reported in children with FN [5]; however, our cohort includes non-neutropenic febrile illness, which could partly explain this difference. Our 13.0% rate of definite bacterial infection was comparable to 11.4–37.0% reported in recent literature [8, 30–32].

Objective clinical features and laboratory investigations at admission did not discriminate well between bacterial and viral infections in our cohort, and neutropenia at admission in our cohort did not significantly change the risk of having a proven/presumed bacterial or viral infection.

Looking at any immunosuppressant use, we did not observe significant associations. Ill appearance was associated with proven/presumed bacterial infection and is known to be a risk factor for bacterial infection [33, 34]. Ill appearance also reduced the risk of having a proven/presumed viral infection. HIV increased the odds of having a proven/presumed bacterial infection; however, we acknowledge that the number of patients with HIV was low and that the results may be skewed by inclusion bias.

We observed considerable variability in empirical antibiotic use across sites, with 29 empirical antibiotics used. This can be partially explained by protocol differences, some centres preferring different glycopeptides, and some children requiring specific antibiotic cover, e.g. for *Burkholderia* in chronic granulomatous disease. A significant proportion of patients, mainly oncology or HSCT patients, were empirically treated with piperacillin-tazobactam with or without teicoplanin, in accordance with guidance on treatment of suspected FN sepsis [35, 36].

There are grounds to assume that a significant proportion of HR children are overtreated with intravenous antibiotics and, similar to the general paediatric population, have self-limiting febrile illness [20, 21, 37]. However, infections remain the main cause of morbidity and mortality in the HR population [7, 38]. Withholding or early discontinuation of antibiotics remains controversial. We do not have sufficient evidence to effectively alter current practice [35, 39, 40]. Immunocompromised children remain frequently hospitalised for intravenous antibiotic treatment, which has a negative impact on patient and family quality of life [17].

We acknowledge that the small proportion of Gambian patients represent different epidemiology and aetiology, and that these patients have less access to biologicals, and specialised diagnostic tests compared to the other sites in this cohort, a known issue in LMIC.

Currently, there is no validated risk stratification tool for this population [41]. In adults, there is a well-used risk stratification for high-risk patients, allowing for short-course, oral, and outpatient parenteral antibiotics that reduced both hospital admission and broad-spectrum antibiotic use [42]. It is not yet proven helpful in children [43].

For patients with T cell deficiencies, seen in certain PID and HSCT patients, viral infections are just as significant as bacterial infections requiring antiviral or immunoglobulin treatment [44, 45]. Both SBI and ‘serious viral infection’ cause significant morbidity and mortality, as demonstrated by the fatal cases in our cohort.

In our cohort, mortality was low at 1.9%, but 9% PICU admission rate demonstrates significant morbidity.

The high percentage of children with no definitive diagnosis demonstrates the need for better diagnostic tests to optimise early, effective, and targeted treatment.

### Strengths and limitations

The study strengths lie in the international and multicentre approach, allowing us to evaluate management across Europe and the Gambia. We collected in-depth patient data, with 28-day follow-up, and included a wide range of immunocompromised children reflecting the clinical spectrum at university hospital EDs. Study limitations lie in the nature of recruitment: episodes in this cohort are biased by referrals and inclusion rates of participating centres across different countries. Therefore, it cannot be judged as a general epidemiological perspective or estimate for proportional incidence rates, nor is management generalisable to other LMIC as the availability of LMIC data in our cohort was low.

The use of experienced paediatricians as reviewers of the assigned final phenotypes potentially induced observer and outcome bias due to intra- and inter-rater differences. This is a known problem and leads to a low inter-rater reliability, as demonstrated in the process of peer reviewing by scientific journals [46] or by assessing performance scores in oncology patients [47]. We mitigated the potentially induced bias to the best of our ability by having two reviewers and an independent consortium episode review in case of disagreement, and using a validated algorithm [27]. Validated scores or algorithms increase inter-rater reliability and have been reported in evaluation of paediatric early warning scores [48].

## Conclusions

Febrile illness and infectious complications remain a significant cause of morbidity and mortality in immunocompromised children. Current management is effective and mortality low, but a significant proportion of children require PICU care. Swift and accurate diagnosis of febrile illness in this population remains challenging. Justifying broad-spectrum intravenous antibiotic treatment of fever for every high-risk patient is costly in terms of drugs, burden of antibiotic resistance, hospitalisation, and costs to families and overburdened healthcare systems. Identifying low-risk febrile patients could reduce hospital admission in this patient population. Future research should focus on development of new rapid clinical decision-making tools and biomarkers targeting immunocompromised paediatric population.

## Supplementary Information

Below is the link to the electronic supplementary material.

Supplementary file1 (DOCX 449 KB)

Supplementary file2 (DOCX 28 KB)

## Abbreviations

ANC: Absolute neutrophil count
CI: Confidence interval
CRP: C-reactive protein
ED: Emergency department
FN: Febrile neutropenia
HIV: Human immunodeficiency virus
HR: High risk
HSCT: Haematopoietic stem cell transplant
IQR: Interquartile range
LMIC: Low- and middle-income country
LOS: Length of in-patient stay
PERFORM: Personalised Risk assessment in Febrile illness to Optimise Real-life Management across the European Union
OR: Odds ratio
PICU: Paediatric intensive care unit
PID: Primary immunodeficiency
SBI: Serious bacterial infection
UK: United Kingdom
