# Correction to: Febrile illness in high-risk children: a prospective, international observational study

**DOI:** 10.1007/s00431-022-04788-y

**Published:** 2023-01-23

**Authors:** Fabian J. S. van der Velden, Gabriella de Vries, Alexander Martin, Emma Lim, Ulrich von Both, Laura Kolberg, Enitan D. Carrol, Aakash Khanijau, Jethro A. Herberg, Tisham De, Rachel Galassini, Taco W. Kuijpers, Federico Martinón-Torres, Irene Rivero-Calle, Clementien L. Vermont, Nienke N. Hagedoorn, Marko Pokorn, Andrew J. Pollard, Luregn J. Schlapbach, Maria Tsolia, Irini Elefhteriou, Shunmay Yeung, Dace Zavadska, Colin Fink, Marie Voice, Werner Zenz, Benno Kohlmaier, Philipp K. A. Agyeman, Effua Usuf, Fatou Secka, Ronald de Groot, Michael Levin, Michiel van der Flier, Marieke Emonts, PERFORM consortium, Michael Levin, Aubrey Cunnington, Tisham De, Jethro Herberg, Myrsini Kaforou, Victoria Wright, Lucas Baumard, Evangelos Bellos, Giselle D’Souza, Rachel Galassini, Dominic Habgood-Coote, Shea Hamilton, Clive Hoggart, Sara Hourmat, Heather Jackson, Ian Maconochie, Stephanie Menikou, Naomi Lin, Samuel Nichols, Ruud Nijman, Oliver Powell, Ivonne Pena Paz, Priyen Shah, Ching-Fen Shen, Ortensia Vito, Clare Wilson, Amina Abdulla, Ladan Ali, Sarah Darnell, Rikke Jorgensen, Sobia Mustafa, Salina Persand, Molly M. Stevens, Nayoung Kim, Eunjung Kim, Katy Fidler, Julia Dudley, Vivien Richmond, Emma Tavliavini, Ching-Fen Shen, Ching-Chuan Liu, Shih-Min Wang, Federico Martinón-Torres, Antonio Salas, Fernando Álvez González, Cristina Balo Farto, Ruth Barral-Arca, María Barreiro Castro, Xabier Bello, Mirian Ben García, Sandra Carnota, Miriam Cebey-López, María José Curras-Tuala, Carlos Durán Suárez, Luisa García Vicente, Alberto Gómez-Carballa, Jose Gómez Rial, Pilar Leboráns Iglesias, Federico Martinón-Torres, Nazareth Martinón-Torres, José María Martinón Sánchez, Belén Mosquera Pérez, Jacobo Pardo-Seco, Lidia Piñeiro Rodríguez, Sara Pischedda, Sara Rey Vázquez, Irene Rivero Calle, Carmen Rodríguez-Tenreiro, Lorenzo Redondo-Collazo, Miguel Sadiki Ora, Antonio Salas, Sonia Serén Fernández, Cristina Serén Trasorras, Marisol Vilas Iglesias, Dace Zavadska, Anda Balode, Arta Bārzdiņa, Dārta Deksne, Dace Gardovska, Dagne Grāvele, Ilze Grope, Anija Meiere, Ieva Nokalna, Jana Pavāre, Zanda Pučuka, Katrīna Selecka, Aleksandra Rudzāte, Dace Svile, Urzula Nora Urbāne, Effua Usuf, Kalifa Bojang, Syed M. A. Zaman, Fatou Secka, Suzanne Anderson, Anna RocaIsatou Sarr, Momodou Saidykhan, Saffiatou Darboe, Samba Ceesay, Umberto D’alessandro, Henriëtte A. Moll, Clementien L Vermont, Dorine M. Borensztajn, Nienke N. Hagedoorn, Chantal Tan, Joany Zachariasse, W Dik, Philipp KA Agyeman, Christoph Berger, Eric Giannoni, Martin Stocker, Klara M Posfay-Barbe, Ulrich Heininger, Sara Bernhard-Stirnemann, Anita Niederer-Loher, Christian R. Kahlert, Giancarlo Natalucci, Christa Relly, Thomas Riedel, Christoph Aebi, Luregn J Schlapbach, Enitan D Carrol, Elizabeth Cocklin, Rebecca Jennings, Joanne Johnston, Aakash Khanijau, Simon Leigh, Nadia Lewis-Burke, Karen Newall, Sam Romaine, Maria Tsolia, Irini Eleftheriou, Maria Tambouratzi, Antonis Marmarinos, Marietta Xagorari, Kelly Syggelou, Colin Fink, Marie Voice, Leo Calvo-Bado, Werner Zenz, Benno Kohlmaier, Nina A. Schweintzger, Manfred G. Sagmeister, Daniela S. Kohlfürst, Christoph Zurl, Alexander Binder, Susanne Hösele, Manuel Leitner, Lena Pölz, Glorija Rajic, Sebastian Bauchinger, Hinrich Baumgart, Martin Benesch, Astrid Ceolotto, Ernst Eber, Siegfried Gallistl, Gunther Gores, Harald Haidl, Almuthe Hauer, Christa Hude, Markus Keldorfer, Larissa Krenn, Heidemarie Pilch, Andreas Pfleger, Klaus Pfurtscheller, Gudrun Nordberg, Tobias Niedrist, Siegfried Rödl, Andrea Skrabl-Baumgartner, Matthias Sperl, Laura Stampfer, Volker Strenger, Holger Till, Andreas Trobisch, Sabine Löffler, Shunmay Yeung, Juan Emmanuel Dewez, Martin Hibberd, David Bath, Alec Miners, Ruud Nijman, Elizabeth Fitchett, Ronald de Groot, Michiel van der Flier, Marien I. de Jonge, Koen van Aerde, Wynand Alkema, Bryan van den Broek, Jolein Gloerich, Alain J. van Gool, Stefanie Henriet, Martijn Huijnen, Ria Philipsen, Esther Willems, G.P.J.M. Gerrits, M. van Leur, J. Heidema, L. de Haan, C.J. Miedema, C. Neeleman, C.C. Obihara, G.A. Tramper-Stranders, Andrew J. Pollard, Rama Kandasamy, Stéphane Paulus, Michael J. Carter, Daniel O’Connor, Sagida Bibi, Dominic F. Kelly, Meeru Gurung, Stephen Thorson, Imran Ansari, David R. Murdoch, Shrijana Shrestha, Zoe Oliver, Marieke Emonts, Emma Lim, Lucille Valentine, Karen Allen, Kathryn Bell, Adora Chan, Stephen Crulley, Kirsty Devine, Daniel Fabian, Sharon King, Paul McAlinden, Sam McDonald, Anne McDonnell, Ailsa Pickering, Evelyn Thomson, Amanda Wood, Diane Wallia, Phil Woodsford, Frances Baxter, Ashley Bell, Mathew Rhodes, Rachel Agbeko, Christine Mackerness, Bryan Baas, Lieke Kloosterhuis, Wilma Oosthoek, Tasnim Arif, Joshua Bennet, Kalvin Collings, Ilona van der Giessen, Alex Martin, Aqeela Rashid, Emily Rowlands, Gabriella de Vries, Fabian van der Velden, Joshua Soon, Lucille Valentine, Mike Martin, Ravi Mistry, Ulrich von Both, Laura Kolberg, Manuela Zwerenz, Judith Buschbeck, Christoph Bidlingmaier, Vera Binder, Katharina Danhauser, Nikolaus Haas, Matthias Griese, Tobias Feuchtinger, Julia Keil, Matthias Kappler, Eberhard Lurz, Georg Muench, Karl Reiter, Carola Schoen, François Mallet, Karen Brengel-Pesce, Alexandre Pachot, Marine Mommert, Marko Pokorn, Mojca Kolnik, Katarina Vincek, Tina Plankar Srovin, Natalija Bahovec, Petra Prunk, Veronika Osterman, Tanja Avramoska, Taco Kuijpers, Ilse Jongerius, J. M. van den Berg, D. Schonenberg, A. M. Barendregt, D. Pajkrt, M. van der Kuip, A. M. van Furth, Evelien Sprenkeler, Judith Zandstra, G. van Mierlo, J. Geissler

**Affiliations:** 1 Paediatric Immunology, Infectious Diseases & Allergy, Great North Children’s Hospital, Newcastle upon Tyne Hospitals NHS Foundation Trust, Newcastle upon Tyne, UK; 2 Translational and Clinical Research Institute, Newcastle University, Newcastle upon Tyne, UK; 3 Department of General Paediatrics, Erasmus MC-Sophia Children’s Hospital, Rotterdam, The Netherlands; 4 Population Health Sciences Institute, Newcastle University, Newcastle upon Tyne, UK; 5 Division Paediatric Infectious Diseases, Dr. Von Hauner Children’s Hospital, University Hospital LMU Munich, Munich, Germany; 6 Institute of Infection, Veterinary and Ecological Sciences, University of Liverpool, Liverpool, UK; 7 Alder Hey Children’s NHS Foundation Trust, Liverpool, UK; 8 Section of Paediatric Infectious Disease, Wright-Fleming Institute, Imperial College London, London, UK; 9 Department of Pediatric Immunology, Rheumatology and Infectious Diseases, Amsterdam University Medical Center, Location Academic Medical Center, University of Amsterdam, Amsterdam, The Netherlands; 10 Pediatrics Department, Translational Pediatrics and Infectious Diseases, Hospital Clínico Universitario de Santiago, Santiago de Compostela, Spain; 11 University Children’s Hospital, University Medical Centre Ljubljana, Ljubljana, Slovenia; 12 Oxford Vaccine Group, Department of Paediatrics, University of Oxford, Oxford, UK; 13 Neonatal and Pediatric Intensive Care Unit, Children’s Research Center, University Children’s Hospital Zürich, University of Zürich, Zurich, Switzerland; 14 2nd Department of Pediatrics, National and Kapodistrian University of Athens, Children’s Hospital ‘P, and A. Kyriakou’, Athens, Greece; 15 Clinical Research Department, Faculty of Infectious and Tropical Disease, London School of Hygiene and Tropical Medicine, London, UK; 16 Department of Pediatrics, Rīgas Stradina Universitāte, Children’s Clinical University Hospital, Riga, Latvia; 17 Micropathology Ltd, University of Warwick, Warwick, UK; 18 Department of Pediatrics and Adolescent Medicine, Division of General Pediatrics, Medical University of Graz, Graz, Austria; 19 Pediatric Infectious Diseases and Immunology, Amalia Children’s Hospital, Radboud University Medical Center, Nijmegen, The Netherlands; 20 NIHR Newcastle Biomedical Research Centre, Newcastle upon Tyne Hospitals NHS Trust and Newcastle University, Newcastle upon Tyne, UK; 21 Department of Pediatrics, Division of Pediatric Infectious Diseases & Immunology, Erasmus MC-Sophia Children’s Hospital, Rotterdam, The Netherlands; 22 Pediatric Infectious Diseases and Immunology, Wilhelmina Children’s Hospital University Medical Center Utrecht, Utrecht, The Netherlands; 23 Department of Pediatrics, Inselspital, Bern University Hospital, University of Bern, Bern, Switzerland; 24 Medical Research Council Unit, Serrekunda, The Gambia; 25 Grupo de Genetica, Vacunas, Infecciones y Pediatria, Instituto de Investigacion Sanitaria de Santiago, Universidad de Santiago, Santiago de Compostela, Spain; 26 Consorcio Centro de Investigacion Biomedicaen Red de Enfermedades Respiratorias (CIBERES), Madrid, Spain

## **Correction to: European Journal of Pediatrics****https://doi.org/10.1007/s00431-022-04642-1**

In the original published version of the above article, the names of members of the PERFORM consortium were not introduced in the authorship section. The names are now properly displayed.

The complete consortium list has been added as Supplementary material to the original article.

The original article has been corrected.

