# Supplementary material for: Febrile illness in high-risk children: a prospective, international observational study
Source: Eur J Pediatr. 2022 Oct 15;182(2):543–54. doi: 10.1007/s00431-022-04642-1 (PMC9899189; doi:10.1007/s00431-022-04642-1)
Supplement: Supplementary file 1 — Supplementary file1 (DOCX 449 KB) [file 431_2022_4642_MOESM1_ESM.docx]

**Supplementary Information**

*Supplementary figure S1:*  *PERFORM Phenotyping Algorithm (Nijman et al. 2021)*


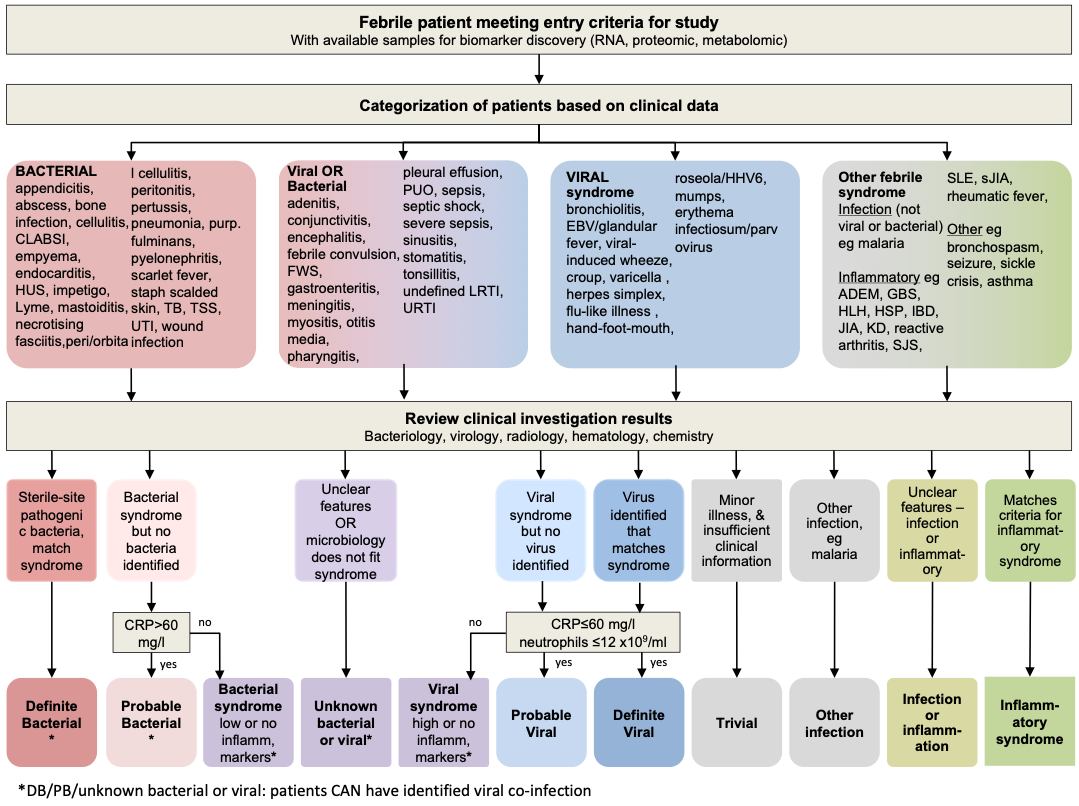


*Supplementary table S1: Cohort demographics at admission by phenotype. GCS: Glasgow Coma Scale; HIV: human immunodeficiency virus; HSCT: haematopoietic stem cell transplant. *age-adjusted vital parameters as per APLS 2017 (>95th centile or <5th centile) Data is presented as N= episodes (%) or median (IQR).*

|  | **Definite bacterial**  **(N=78)** | **Probable bacterial**  **(N=67)** | **Bacterial syndrome**  **(N=29)** | **Unknown bacterial or viral infection**  **(N=190)** | **Viral syndrome**  **(N=23)** | **Probable viral**  **(N=49)** | **Definite viral**  **(N=55)** | **Trivial illness**  **(N=5)** | **Other infection**  **(N=3)** | **Uncertain infection or inflammation**  **(N=26)** | **Inflammatory syndrome**  **(N=26)** | **Other cause of illness**  **(N=48)** |
| --- | --- | --- | --- | --- | --- | --- | --- | --- | --- | --- | --- | --- |
| Male | 48 (61.5%) | 37 (55.2%) | 16 (55.2%) | 107 (56.3%) | 13 (56.5%) | 23 (46.9%) | 34 (61.8%) | 3 (60.0%) | 3 (100%) | 20 (76.9%) | 12 (46.2%) | 27 (56.3%) |
| Age (years) | 7.4 (3.3-13.5) | 8.2 (3.6-12.8) | 7.6 (3.5-12.9) | 6.5 (4.1-11.7) | 9.1 (3.3-15.0) | 7.2 (4.4-11.7) | 6.7 (4.5-11.0) | 7.8 (5.1-16.0) | 9.0 (0.2-9.0) | 9.8 (3.1-14.9) | 11.8 (4.7-15.1) | 10.3 (4.7-15.0) |
| HSCT patient | 9 (11.5%) | 4 (6.0%) | 2 (6.9%) | 15 (7.9%) | 3 (13.0%) | 2 (4.1%) | 14 (25.5%) | 0 (0.0%) | 1 (33.3%) | 8 (30.8%) | 5 (19.2%) | 6 (12.5%) |
| **Underlying condition** |  |  |  |  |  |  |  |  |  |  |  |  |
| Malignancy | 45 (57.7%) | 38 (56.7%) | 15 (51.7%) | 150 (78.9%) | 11 (47.8%) | 28 (57.1%) | 30 (54.5%) | 3 (60.0%) | 1 (33.3%) | 8 (30.8%) | 0 (0.0%) | 25 (52.1%) |
| Haematological disease | 6 (7.7%) | 12 (17.9%) | 3 (10.3%) | 19 (10.0%) | 2 (8.7%) | 6 (12.2%) | 11 (20.0%) | 1 (20.0%) | 0 (0.0%) | 4 (15.4%) | 1 (3.8%) | 14 (29.2%) |
| Inflammatory syndromes | 4 (5.1%) | 0 (0.0%) | 3 (10.3%) | 5 (2.6%) | 2 (8.7%) | 3 (6.1%) | 4 (7.3%) | 1 (20.0%) | 0 (0.0%) | 7 (26.9%) | 17 (65.4%) | 1 (2.1%) |
| Primary immunodeficiency | 2 (2.6%) | 6 (9.0%) | 2 (6.9%) | 8 (4.2%) | 3 (13.0%) | 6 (12.2%) | 5 (9.1%) | 0 (0.0%) | 0 (0.0%) | 5 (19.2%) | 8 (30.8%) | 2 (4.2%) |
| Solid organ transplant | 10 (12.8%) | 7 (10.4%) | 2 (6.9%) | 3 (1.6%) | 2 (8.7%) | 1 (2.0%) | 1 (1.8%) | 0 (0.0%) | 0 (0.0%) | 1 (3.8%) | 0 (0.0%) | 3 (6.3%) |
| HIV | 2 (2.6%) | 2 (3.0%) | 2 (6.9%) | 0 (0.0%) | 0 (0.0%) | 0 (0.0%) | 0 (0.0%) | 0 (0.0%) | 1 (33.3%) | 0 (0.0%) | 0 (0.0%) | 0 (0.0%) |
| Nephrotic syndrome | 2 (2.6%) | 1 (1.5%) | 0 (0.0%) | 1 (0.5%) | 0 (0.0%) | 0 (0.0%) | 1 (1.8%) | 0 (0.0%) | 0 (0.0%) | 0 (0.0%) | 0 (0.0%) | 1 (2.1%) |
| Cystic fibrosis | 0 (0.0%) | 1 (1.5%) | 1 (3.4%) | 2 (1.1%) | 0 (0.0%) | 0 (0.0%) | 1 (1.8%) | 0 (0.0%) | 0 (0.0%) | 0 (0.0%) | 0 (0.0%) | 0 (0.0%) |
| Short bowel syndrome | 2 (2.6%) | 0 (0.0%) | 0 (0.0%) | 1 (0.5%) | 0 (0.0%) | 1 (2.0%) | 0 (0.0%) | 0 (0.0%) | 0 (0.0%) | 0 (0.0%) | 0 (0.0%) | 0 (0.0%) |
| Other conditions | 5 (6.4%) | 0 (0.0%) | 1 (3.4%) | 1 (0.5%) | 3 (13.0%) | 4 (8.2%) | 2 (3.6%) | 0 (0.0%) | 1 (33.3%) | 0 (0.0%) | 0 (0.0%) | 2 (4.2%) |
| **Clinical features** |  |  |  |  |  |  |  |  |  |  |  |  |
| Ill appearance | 43 (55.1%) | 34 (50.7%) | 6 (20.7%) | 39 (20.5%) | 9 (39.1%) | 9 (18.4%) | 8 (14.5%) | 0 (0.0%) | 1 (33.3%) | 5 (19.2%) | 4 (15.4%) | 18 (37.5%) |
| Lifesaving intervention required | 14 (17.9%) | 9 (13.4%) | 3 (10.3%) | 9 (4.7%) | 4 (17.4%) | 2 (4.1%) | 2 (3.6%) | 0 (0.0%) | 1 (33.3%) | 4 (15.4%) | 1 (3.8%) | 5 (10.4%) |
| Diarrhoea | 5 (6.4%) | 7 (10.4%) | 2 (6.9%) | 9 (4.7%) | 1 (4.3%) | 4 (8.2%) | 5 (9.1%) | 0 (0.0%) | 0 (0.0%) | 5 (19.2%) | 5 (19.2%) | 2 (4.2%) |
| Increased work of breathing | 3 (3.8%) | 7 (10.4%) | 1 (3.4%) | 6 (3.2%) | 4 (17.4%) | 2 (4.1%) | 4 (7.3%) | 0 (0.0%) | 1 (33.3%) | 4 (15.4%) | 1 (3.8%) | 3 (6.3%) |
| Vomiting | 8 (10.3%) | 3 (4.5%) | 1 (3.4%) | 5 (2.6%) | 3 (13.0%) | 2 (4.1%) | 1 (1.8%) | 0 (0.0%) | 1 (33.3%) | 2 (7.7%) | 1 (3.8%) | 1 (2.1%) |
| Non-blanching rash | 2 (2.6%) | 3 (4.5%) | 1 (3.4%) | 2 (1.1%) | 1 (4.3%) | 0 (0.0%) | 1 (1.8%) | 0 (0.0%) | 0 (0.0%) | 3 (11.5%) | 1 (3.8%) | 1 (2.1%) |
| Clinical dehydration | 2 (2.6%) | 3 (4.5%) | 1 (3.4%) | 3 (1.6%) | 1 (4.3%) | 3 (6.1%) | 0 (0.0%) | 0 (0.0%) | 0 (0.0%) | 1 (3.8%) | 0 (0.0%) | 1 (2.1%) |
| Seizures | 2 (2.6%) | 2 (3.0%) | 0 (0.0%) | 2 (1.1%) | 0 (0.0%) | 0 (0.0%) | 0 (0.0%) | 0 (0.0%) | 0 (0.0%) | 1 (3.8%) | 0 (0.0%) | 1 (2.1%) |
| Meningism | 1 (1.3%) | 0 (0.0%) | 0 (0.0%) | 0 (0.0%) | 1 (4.3%) | 0 (0.0%) | 0 (0.0%) | 0 (0.0%) | 0 (0.0%) | 0 (0.0%) | 0 (0.0%) | 1 (2.1%) |
| **Vital parameters, age adjusted*** |  |  |  |  |  |  |  |  |  |  |  |  |
| Tachypnoea | 10 (12.8%) | 19 (28.4%) | 3 (10.3%) | 16 (8.4%) | 3 (13.0%) | 6 (12.2%) | 6 (10.9%) | 0 (0.0%) | 1 (33.3%) | 5 (19.2%) | 3 (11.5%) | 7 (14.6%) |
| Bradypnoea | 2 (2.6%) | 2 (3.0%) | 1 (3.4%) | 2 (1.1%) | 0 (0.0%) | 1 (2.0%) | 0 (0.0%) | 0 (0.0%) | 0 (0.0%) | 1 (3.8%) | 0 (0.0%) | 0 (0.0%) |
| Low saturation (<94% in air) | 0 (0.0%) | 10 (14.9%) | 1 (3.4%) | 9 (4.7%) | 4 (17.4%) | 0 (0.0%) | 4 (7.3%) | 0 (0.0%) | 1 (33.3%) | 3 (11.5%) | 1 (3.8%) | 6 (12.5%) |
| Tachycardia | 32 (41.0%) | 31 (46.3%) | 6 (20.7%) | 63 (33.2%) | 9 (39.1%) | 15 (30.6%) | 11 (20.0%) | 1 (20.0%) | 1 (33.3%) | 4 (15.4%) | 7 (26.9%) | 9 (18.8%) |
| Bradycardia | 1 (1.3%) | 0 (0.0%) | 0 (0.0%) | 1 (0.5%) | 0 (0.0%) | 0 (0.0%) | 0 (0.0%) | 0 (0.0%) | 0 (0.0%) | 0 (0.0%) | 0 (0.0%) | 0 (0.0%) |
| Hypotension | 7 (9.0%) | 7 (10.4%) | 0 (0.0%) | 15 (7.9%) | 3 (13.0%) | 3 (6.1%) | 5 (9.1%) | 0 (0.0%) | 0 (0.0%) | 1 (3.8%) | 3 (11.5%) | 3 (6.3%) |
| Hypertension | 27 (34.6%) | 22 (32.8%) | 7 (24.1%) | 43 (22.6%) | 8 (34.8%) | 14 (28.6%) | 14 (25.5%) | 3 (60.0%) | 1 (33.3%) | 9 (34.6%) | 10 (38.5%) | 21 (43.8%) |
| Prolonged capillary refill time (>2 seconds) | 3 (4.5%) | 5 (8.6%) | 8 (27.6%) | 5 (2.6%) | 1 (6.3%) | 1 (2.7%) | 0 (0.0%) | 0 (0.0%) | 0 (0.0%) | 1 (5.3%) | 0 (0.0%) | 0 (0.0%) |
| Decreased consciousness (AVPU <A, GCS <= 13) | 0 (0.0%) | 2 (3.0%) | 0 (0.0%) | 2 (1.1%) | 0 (0.0%) | 0 (0.0%) | 0 (0.0%) | 0 (0.0%) | 0 (0.0%) | 0 (0.0%) | 1 (3.8%) | 0 (0.0%) |
| Fever (documented/history => 38.0°C) | 76 (97.4%) | 59 (88.1%) | 20 (69.0%) | 178 (93.7%) | 22 (95.7%) | 44 (89.8%) | 49 (89.1%) | 3 (60.0%) | 3 (100%) | 21 (80.8%) | 21 (80.8%) | 32 (66.7%) |
| **Blood investigations** |  |  |  |  |  |  |  |  |  |  |  |  |
| Neutropenia | 27 (34.6%) | 27 (40.4%) | 7 (24.1%) | 101 (53.2%) | 5 (21.7%) | 10 (20.8%) | 18 (32.7%) | 1 (20.0%) | 0 (0.0%) | 5 (20.0%) | 2 (7.7%) | 9 (18.8%) |
| Lymphopenia | 35 (54.7%) | 33 (57.9%) | 7 (29.2%) | 89 (46.8%) | 11 (47.8%) | 20 (48.8%) | 32 (62.7%) | 3 (60.0%) | 1 (33.3%) | 13 (68.4%) | 7 (29.2%) | 14 (34.1%) |
| **Immunomodulating drug use** |  |  |  |  |  |  |  |  |  |  |  |  |
| Biologicals | 4 (5.1%) | 1 (1.5%) | 1 (3.4%) | 6 (3.2%) | 2 (8.7%) | 2 (4.1%) | 1 (1.8%) | 1 (20.0%) | 0 (0.0%) | 4 (15.4%) | 8 (30.8%) | 4 (8.3%) |
| Ciclosporin | 5 (6.4%) | 4 (6.0%) | 1 (3.4%) | 5 (2.6%) | 1 (4.3%) | 1 (2.0%) | 7 (12.7%) | 0 (0.0%) | 1 (33.3%) | 4 (15.4%) | 3 (11.5%) | 3 (6.3%) |
| Colchicine | 0 (0.0%) | 0 (0.0%) | 0 (0.0%) | 0 (0.0%) | 0 (0.0%) | 1 (2.0%) | 0 (0.0%) | 0 (0.0%) | 0 (0.0%) | 0 (0.0%) | 0 (0.0%) | 0 (0.0%) |
| Immunoglobulin | 3 (3.8%) | 5 (7.5%) | 1 (3.4%) | 8 (4.2%) | 1 (4.3%) | 2 (4.1%) | 7 (12.7%) | 0 (0.0%) | 0 (0.0%) | 6 (23.1%) | 4 (15.4%) | 2 (4.2%) |
| Methotrexate | 11 (14.1%) | 14 (20.9%) | 6 (20.7%) | 45 (23.7%) | 4 (17.4%) | 13 (26.5%) | 15 (27.3%) | 1 (20.0%) | 0 (0.0%) | 2 (7.7%) | 5 (19.2%) | 2 (4.2%) |
| Steroids | 27 (34.6%) | 10 (14.9%) | 4 (13.8%) | 35 (18.4%) | 6 (26.1%) | 8 (16.3%) | 7 (12.7%) | 0 (0.0%) | 0 (0.0%) | 7 (26.9%) | 11 (42.3%) | 7 (14.6%) |
| Tacrolimus | 10 (12.8%) | 6 (9.0%) | 2 (6.9%) | 3 (1.6%) | 2 (8.7%) | 1 (2.0%) | 2 (3.6%) | 0 (0.0%) | 0 (0.0%) | 2 (7.7%) | 0 (0.0%) | 4 (8.3%) |
| Other immunomodulating drug | 35 (44.9%) | 30 (44.8%) | 13 (44.8%) | 76 (40.0%) | 12 (52.2%) | 27 (55.1%) | 26 (47.3%) | 4 (80.0%) | 0 (0.0%) | 9 (34.6%) | 9 (34.6%) | 21 (43.8%) |

*Supplementary Table S2: Detailed underlying diagnoses by episode*

| **Underlying diagnosis** | **All (N=599)** | **Proven/presumed bacterial (N=174)** | **Proven/presumed viral (N=127)** |
| --- | --- | --- | --- |
| **Any malignancy** | **354** | **98** | **68** |
| **Haematological malignancy** | **227** | **61** | **45** |
| ALL | 155 | 38 | 39 |
| AML | 24 | 11 | 1 |
| Lymphoma | 10 | 2 | 1 |
| Non-Hodgkin lymphoma | 8 | 3 | 1 |
| Langerhans cell histiocytosis | 7 | 1 | 1 |
| Aplastic anaemia | 5 | 1 | 0 |
| Burkitt lymphoma | 5 | 0 | 1 |
| Hodgkin lymphoma | 3 | 0 | 0 |
| JMML | 2 | 1 | 1 |
| Blastic Plasmacytoid Dendritic Cell Neoplasm | 1 | 1 | 0 |
| Fanconi anaemia | 1 | 1 | 0 |
| Histiocytosis | 1 | 0 | 0 |
| Leukaemia | 1 | 0 | 0 |
| Leukaemia-like | 1 | 1 | 0 |
| Myelodysplastic syndrome | 1 | 0 | 0 |
| Myeloblastoma | 1 | 1 | 0 |
| Schwachman-Diamond syndrome | 1 | 0 | 1 |
| **Solid malignancy** | **90** | **27** | **14** |
| Osteosarcoma | 21 | 11 | 3 |
| Ewing sarcoma | 20 | 4 | 2 |
| Neuroblastoma | 17 | 3 | 4 |
| Wilms tumour | 8 | 2 | 1 |
| Rhabdomyosarcoma | 7 | 2 | 1 |
| PNET | 4 | 2 | 0 |
| Rhabdoid tumour | 3 | 1 | 1 |
| Sarcoma | 2 | 0 | 1 |
| Synovial sarcoma | 2 | 0 | 0 |
| Germ cell tumour | 1 | 0 | 1 |
| Hepatoblastoma | 1 | 1 | 0 |
| Myeloid sarcoma | 1 | 0 | 0 |
| Nephroblastoma | 1 | 0 | 0 |
| Peripheral Nerve Sheath Tumour | 1 | 1 | 0 |
| Small blue round cell tumour | 1 | 0 | 0 |
| **Brain/CNS malignancy** | **37** | **10** | **9** |
| Medulloblastoma | 10 | 3 | 4 |
| Glioma | 8 | 2 | 3 |
| Ependymoma | 7 | 3 | 0 |
| Atypical Teratoid Rhabdoid Tumour | 3 | 1 | 0 |
| Retinoblastoma | 3 | 0 | 1 |
| Astrocytoma | 1 | 1 | 0 |
| Choroid plexus tumour | 1 | 0 | 0 |
| Endodermic sinus tumour | 1 | 0 | 0 |
| Glioblastoma | 1 | 0 | 0 |
| Pineoblastoma | 1 | 0 | 1 |
| Posterior fossa tumour | 1 | 0 | 0 |
| **Haematological disease** | **79** | **21** | **19** |
| Sickle cell disease | 44 | 13 | 7 |
| Autoimmune neutropenia | 8 | 1 | 2 |
| Beta thalassaemia | 6 | 0 | 2 |
| Asplenia | 3 | 1 | 1 |
| Hereditary spherocytosis | 3 | 1 | 1 |
| Haemolytic anaemia | 2 | 0 | 1 |
| Neutropenia e.c.i | 2 | 1 | 1 |
| Autoimmune granulocytopenia | 1 | 0 | 1 |
| Autoimmune haemolytic anaemia | 1 | 0 | 1 |
| Chronic neutropenia | 1 | 0 | 0 |
| Congenital neutropenia | 1 | 0 | 1 |
| Cyclic neutropenia | 1 | 1 | 0 |
| Diamond-Blackfan anaemia | 1 | 0 | 0 |
| Henoch-Schonlein purpura | 1 | 1 | 0 |
| RALD-Ras associated autoimmuno leukoproliferative disease | 1 | 1 | 0 |
| Spherocytosis | 1 | 0 | 1 |
| Thrombocytopenia e.c.i. | 1 | 0 | 0 |
| Thrombotic thrombocytopenic purpura | 1 | 1 | 0 |
| **Inflammatory** | **47** | **7** | **7** |
| Crohn disease | 14 | 2 | 1 |
| Ulcerative colitis | 8 | 2 | 2 |
| sJIA | 7 | 0 | 1 |
| JIA | 4 | 0 | 1 |
| Granulomatosis with polyangiitis | 2 | 0 | 2 |
| Haemophagocytic lymphohistiocytosis | 2 | 0 | 0 |
| SLE | 2 | 0 | 0 |
| Autoimmune enteropathy | 1 | 0 | 0 |
| Autoimmune hepatitis | 1 | 0 | 1 |
| Autoinflammatory syndrome e.c.i. | 1 | 1 | 0 |
| Behcet disease | 1 | 0 | 0 |
| Megacystis-microcolon-intestinal hypoperistalsis syndrome | 1 | 1 | 0 |
| Optic neuritis | 1 | 1 | 0 |
| Polyarteritis nodosum | 1 | 0 | 0 |
| Polychondritis | 1 | 0 | 1 |
| **PID** | **47** | **10** | **14** |
| Severe combined immunodeficiency | 7 | 1 | 2 |
| Chronic granulomatous disease | 4 | 1 | 1 |
| STAT1-GOF | 4 | 0 | 2 |
| CD40 Ligand Deficiency | 3 | 1 | 0 |
| Combined immunodeficiency | 2 | 0 | 0 |
| Hypogammaglobulinaemia | 2 | 1 | 1 |
| IgA deficiency | 2 | 0 | 1 |
| IKAROS/IKZF1 deficiency | 2 | 0 | 1 |
| MHC-II deficiency | 2 | 0 | 0 |
| Wiskott Aldrich syndrome | 2 | 0 | 1 |
| Activated P13 Kinase Delta Syndrome | 1 | 0 | 0 |
| Arteriovenous malformation with specific antibody synthesis defect | 1 | 1 | 0 |
| Ataxia telangiectasia | 1 | 1 | 0 |
| Complement c3 deficiency | 1 | 0 | 1 |
| Chediak-Higashi syndrome | 1 | 0 | 0 |
| GATA2 deficiency | 1 | 1 | 0 |
| IgA/IgG subclass deficiency | 1 | 0 | 0 |
| IPEX syndrome | 1 | 1 | 0 |
| SPINK1 mutation | 1 | 0 | 0 |
| Methyl-CpG Binding protein 2 Xq28 duplication syndrome | 1 | 1 | 0 |
| Mowat-Wilson syndrome | 1 | 1 | 0 |
| Omenn syndrome | 1 | 0 | 1 |
| PAX2 mutation | 1 | 0 | 1 |
| Protein C kinase delta immunodeficiency | 1 | 0 | 1 |
| TRAC deficiency | 1 | 0 | 0 |
| Undiagnosed immunodeficiency | 1 | 0 | 1 |
| X-linked agammaglobulinaemia | 1 | 0 | 0 |
| **Solid organ transplant** | **30** | **19** | **4** |
| Kidney | 23 | 15 | 4 |
| Liver | 4 | 3 | 0 |
| Heart | 2 | 1 | 0 |
| Kidney and liver | 1 | 0 | 0 |
| **HIV** | **8** | **6** | **0** |
| **Nephrotic syndrome** | **6** | **3** | **1** |
| **Cystic fibrosis** | **5** | **2** | **1** |
| **Short bowel syndrome** | **4** | **2** | **1** |
| **Other** | **19** | **6** | **9** |
| Medication induced | 5 | 3 | 2 |
| Sarcoidosis | 2 | 0 | 1 |
| Atypical Haemolytic Uraemic Syndrome | 1 | 0 | 1 |
| Bronchopulmonary dysplasia | 1 | 0 | 0 |
| c3 glomerulopathy | 1 | 0 | 1 |
| Camptodactyly-Arthropathy-Coxa-Vara-Pericarditis syndrome | 1 | 0 | 1 |
| Colitis e.c.i. | 1 | 1 | 0 |
| Congenital adrenal hyperplasia | 1 | 0 | 1 |
| Dyskeratosis congenita | 1 | 0 | 0 |
| Hurler syndrome | 1 | 1 | 0 |
| Lymphatic malformations | 1 | 1 | 0 |
| Osteopetrosis | 1 | 0 | 0 |
| Sclerodermia | 1 | 0 | 1 |
| Tuburous sclerosis | 1 | 0 | 1 |

*For the Gambian patients, 7 had sickle cell disease, and 1 HIV as underlying condition*

*Supplementary Table S3: Detailed empirical antimicrobials given and AwARe classification. Number exceeds total of episodes as multiple antimicrobials could have been started empirically, as well as multiple antibiotics from the same antibiotic class.*

| **Antimicrobials by group** | **N= episodes** | **%** | **AWaRE** |
| --- | --- | --- | --- |
| **1st generation cephalosporins** | **3** | 0.5 |  |
| Cefalexin | 2 |  | Access |
| Cefazolin | 1 |  | Access |
| **2nd generation cephalosporins** | **15** | 2.5 |  |
| Cefuroxime | 15 |  | Watch |
| **3rd generation cephalosporins** | **106** | 17.7 |  |
| Ceftriaxone | 44 |  | Watch |
| Ceftazidime | 36 |  | Watch |
| Cefotaxime | 26 |  | Watch |
| **4th generation cephalosporins** | **71** | 11.9 |  |
| Cefepim | 71 |  | Watch |
| **Aminoglycosides** | **109** | 18.2 |  |
| Amikacin | 63 |  | Access |
| Gentamicin | 44 |  | Access |
| Tobramycin | 2 |  | Watch |
| **Amphenicols** | **2** | 0.3 |  |
| Chloramphenicol | 2 |  | Access |
| **Carbapenems** | **41** | 6.8 |  |
| Meropenem | 41 |  | Watch |
| **DHFR inhibitors** | **9** | 1.5 |  |
| Trimethoprim/sulphamethoxazole | 6 |  | Access |
| Trimethoprim | 3 |  | Access |
| **Fluroquinolones** | **15** | 2.5 |  |
| Ciprofloxacin | 15 |  | Watch |
| **Glycopeptides** | **138** | 23.0 |  |
| Teicoplanin | 115 |  | Watch |
| Vancomycin | 26 |  | Watch |
| **Imidazoles** | **19** | 3.2 |  |
| Metronidazole | 19 |  | Access |
| **Lincosamides** | **10** | 1.7 |  |
| Clindamycin | 10 |  | Access |
| **Macrolides** | **29** | 4.8 |  |
| Clarithromycin | 23 |  | Watch |
| Azithromycin | 8 |  | Watch |
| **Oxazolidinones** | **1** | 0.2 |  |
| Linezolid | 1 |  | Reserve |
| **Penicillins** | **257** | 42.9 |  |
| Piperacillin/tazobactam | 197 |  | Watch |
| Amoxicillin/clavulanic acid | 32 |  | Access |
| Amoxicillin | 18 |  | Access |
| Ampicillin | 6 |  | Access |
| Flucloxacillin | 6 |  | Access |
| Phenoxymethylpenicillin | 2 |  | Access |
| Cloxacillin | 1 |  | Access |
| **Other antibiotics** | **12** | 2.0 |  |
| **Antivirals** | **37** | 6.2 |  |
| Aciclovir | 22 |  |  |
| Oseltamavir | 12 |  |  |
| Foscarnet | 1 |  |  |
| Ganciclovir | 1 |  |  |
| Valaciclovir | 1 |  |  |
| **Antifungals** | **23** | 3.8 |  |
| Fluconazole | 10 |  |  |
| Amphotericin | 7 |  |  |
| Caspofungin | 4 |  |  |
| Clotrimazole | 2 |  |  |
| **No antimicrobials** | **77** | 12.9 |  |

*For the Gambian patients: 4 episodes were started on amoxicillin, 2 on ampicillin and gentamicin, and 2 had no empirical antibiotics started*

*Supplementary Table S4: Detailed clinical syndromes by episode (n=599)*

| **Clinical syndrome** | **N= Episodes** |
| --- | --- |
| **Undifferentiated fever** | **144** |
| Fever without source | 118 |
| Fever unknown origin | 26 |
| **Upper respiratory tract infection** | **93** |
| URTI (non-specific) | 68 |
| Stomatitis | 6 |
| Tonsillitis | 6 |
| Otitis media | 5 |
| Pharyngitis | 5 |
| Abscess | 1 |
| Laryngitis subglottica | 1 |
| Otitis externa | 1 |
| **Sepsis syndromes** | **62** |
| CLABSI | 40 |
| Bacteraemia | 18 |
| Septic shock | 2 |
| Endocarditis | 1 |
| Infected thrombus | 1 |
| **Lower respiratory tract infection** | **53** |
| Pneumonia | 30 |
| Undefined LRTI | 17 |
| Bronchiolitis | 3 |
| CF exacerbation | 1 |
| Pleural effusion | 1 |
| Viral induced wheeze | 1 |
| **Viral pathogen syndromes** | **44** |
| Flu(-like) illness | 17 |
| Herpes Simplex virus stomatitis | 4 |
| Roseola | 4 |
| Varicella | 4 |
| Viral infection (unspecified) | 4 |
| Adenoviraemia | 3 |
| Epstein-Barr virus | 2 |
| Herpes Simplex virus | 2 |
| Parvovirus B19 | 2 |
| **Febrile neutropenia (only)** | **42** |
| **Gastrointestinal infection** | **25** |
| Diarrhoea (moderate/severe) | 17 |
| Gastroenteritis | 6 |
| Colitis | 1 |
| Enterocolitis | 1 |
| **Urinary tract infection** | **24** |
| UTI | 15 |
| Pyelonephritis | 9 |
| **Soft tissue infection** | **17** |
| Cellulitis | 6 |
| Wound infection | 5 |
| Abscess (skin) | 3 |
| Impetigo | 1 |
| Lymphadenitis | 1 |
| Periorbital cellulitis | 1 |
| **CNS infection** | **6** |
| Meningitis | 4 |
| Cerebrospinal fistula | 1 |
| Encephalitis | 1 |
| **Surgical infection** | **5** |
| Abscess (intra-abdominal) | 1 |
| Appendicitis | 1 |
| Cholangitis | 1 |
| Mesenteric lymphadenitis | 1 |
| Peritonitis | 1 |
| **Muskuloskeletal infection** | **1** |
| Myositis | 1 |
| **Other infections** | **2** |
| Conjunctivitis | 2 |
| **Other not infections** | **81** |
| Sickle crisis | 12 |
| ALL | 11 |
| sJIA | 6 |
| Crohn exacerbation | 5 |
| Abdominal pain e.c.i. | 4 |
| Engrafting | 4 |
| AML | 3 |
| Drug induced fever | 3 |
| Ulcerative colitis exacerbation | 3 |
| Graft-versus-host disease | 2 |
| Inflammatory syndrome e.c.i. | 2 |
| Lymphoma | 2 |
| Pancreatitis | 2 |
| Seizures | 2 |
| SLE | 2 |
| Toxicodermy | 2 |
| Arhtralgia | 1 |
| Back pain e.c.i | 1 |
| BCG-IRIS | 1 |
| Behcet disease | 1 |
| Cholelithiasis | 1 |
| Chronic constipation | 1 |
| Dermatitis | 1 |
| Haematoma post-biopsy | 1 |
| Hodgkin lymphoma | 1 |
| Irritable bowel syndrome | 1 |
| Immune reconstitution syndrome | 1 |
| HLH | 1 |
| JIA | 1 |
| Leukaemia | 1 |
| Myelodysplastic syndrome | 1 |
| Non-Hodgkin lymphoma | 1 |
| Rhabdomysarcoma | 1 |

*For the Gambian patients: 4 had fever without source, 2 pneumonia,
1 bacteraemia, and 1 an UTI*
